# Development of Scaffold Based on Buriti Oil and PEG-400 at Low Temperature

**DOI:** 10.1021/acsomega.5c05717

**Published:** 2025-12-17

**Authors:** Fernando da S Reis, Thátila Wanessa V de Sousa, Amanda Furtado Luna, Wanderson Gabriel G de Melo, Napoleão M A Neto, Mahendra Rai, Ana Cristina Fialho, Juliani Caland, Ettore Ferrari Júnior, Fabianne Lima, Janildo Lopes Magalhães, José Milton E de Matos

**Affiliations:** † Department of Chemistry, Postgraduate Program of Chemistry, 67823 Federal University of Piauí (UFPI), Teresina 64049-550, Brazil; ‡ Integrated Nucleus of Morphology and Stem Cell Research (NUPCelt), Postgraduate Program in Technologies Applied to Animals of Regional Interest, Federal University of Piauí, Teresina 64049-550, Brazil; § Department of Biotechnology, Sant Gadge Baba Amravati University, Amravati 444602, India; ∥ Department of Pathology and Dental Clinic, Center of Health Sciences, Federal University of Piaui-UFPI, Teresina 64049-550, Brazil; ⊥ Institute of Physics University of Brasília, Brasília 70919-970, Brazil; # Forensic Analysis Laboratory, Criminalistics Institute of the Federal District, Brasília 70610-907, Brazil; ∇ Faculty of Dentistry, Universidade de São Paulo, São Paulo 05508-000, Brazil

## Abstract

Bone mass loss due to trauma or disease is a growing problem, requiring biocompatible and structurally stable materials for bone regeneration. In this study, interconnected porous scaffolds were developed from monoacylglyceride (MAG) derived from buriti oil (OB), combined with PEG-400, to examine the feasibility of MAG/PEG-400 as a biomedical scaffold to overcome the disadvantages of traditional implants. Fourier transform infrared (FTIR) analyses confirmed the formation of the scaffolds (Sc1 and Sc2), and scanning electron microscopy (SEM) images revealed porous structures with aver-age pore sizes of 248 and 258 μm, ideal for bone regeneration. The presence of β-carotene in the scaffolds was evidenced by UV–vis, and their thermal stability exceeded 200 °C. MTT assays indicated cell viability comparable to the control, while hemolysis tests revealed higher hemolytic activity in Sc1, possibly due to PEG-400. These OB-based polyurethanes stand out as a promising innovation, providing a suitable cellular microenvironment for bone regeneration and representing a significant contribution to sustainable biomaterials in tissue engineering (TE).

## Graphical abstract

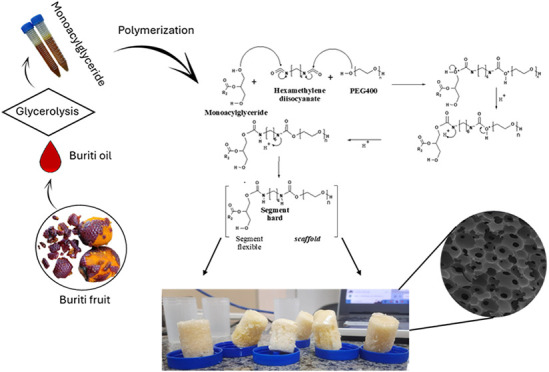

## Introduction

Regenerative medicine has introduced new techniques to treat diseases and regenerate damaged tissues that cannot repair themselves on their own.^1–3^ Bone is a multifunctional and dynamic organ, essential for the support and protection of the human body. Although it is a tissue capable of regenerating and repairing itself, this capacity has limits. Serious injuries, resulting from accidents, illnesses, tumors or other causes, require external intervention and assistance to stimulate bone regeneration in the affected region.^4–6^

Standard practices for bone tissue regeneration include autografts, allografts, and artificial bone substitutes. Despite being effective, grafts face several significant limitations, including graft rejec-tion, the need for additional surgeries, size and shape limitations, and associated discomfort, pain, and morbidity.^7–10^ Tissue engineering (TE) provides an approach to develop polymer-based substitutes, along with bioactive ingredients can provide similar structures and functions to the target tissue through increased cell/structure interactions that can promote osteogenesis.^11–14^

From this perspective, poly­(ethylene glycol) 400 (PEG 400) stands out for being a polymer approved by the FDA due to its biocompatibility, hydrophilicity and nontoxicity, characteristics that make it widely used in human medicine. In addition to playing a crucial role in tissue engineering, it contributes to the generation and expansion of pores in scaffolds, facilitating cell adhesion, growth and proliferation. Furthermore, it has low glass transition temperatures (0 °C) and melting temperatures (70 °C) and is valued for its biodegradability and lack of immunogenicity. Studies highlight its benefits in obtaining scaffolds.^15–20^ Polymers with a low glass transition and melting temperature tend to be more compatible, and can be designed to degrade slowly over time, which is desirable for applications in bone tissue.^19,21–24^

Scaffold is a perfectly designed three-dimensional structure with interconnected pores, which as-sociated with osteogenic materials can provide a microenvironment favorable to adhesion, cell prolif-eration, providing mechanical support that promote cellular functions such as osteoconduction proper-ties (facilitates bone growth on it) and osteoinduction (stimulates the formation of new bone tissue) being an approach with great therapeutic potential in ET.^25–28^

Several studies have demonstrated that the incorporation of bioactive molecules, such as active ingredients derived from plants and biomacromolecules, in the design of scaffolds for ET can induce the process of osteogenesis.^29–31^ These molecules have aroused great interest in the scientific community, as epidemiological evidence points to the benefits of their consumption in preventing various human diseases.^32,33^ Various vegetable oils (soybean, rapeseed, castor and palm), based on triacylglycerides (TAG) of biological origin, are used in the production of high molecular weight polyurethanes (PU), standing out for their advantageous properties, such as natural antioxidants, biocompatibility, biodegradability and lack of toxicity. Furthermore, they are sources of bioactive mole-cules.^27,34,35^ To be widely used as a raw material for chemical synthesis, vegetable oils need to be chemically modified to introduce hydroxyl groups into their structures to generate polyols, precursors, in PU synthesis.^36–39^

In our previous studies, a polyol, MAG, based on OB, was synthesized by a low temperature glycerolysis reaction and was used in this work as a precursor for scaffold synthesis.^40^ In search of natural regulators of osteogenesis, we investigated MAG from OB due to its biological properties and bioactive compounds. These compounds, combined with PEG 400, were used to design alternating segments of rigidity and flexibility, playing a crucial role in the morphology and properties of the scaffold.^41–47^

Buriti (*Mauritia flexuosa* L.) is a palm tree native to several regions of Latin America, whose fruit is a rich source of bioactive compounds, such as carotenoids and phenolic compounds. The oil extracted from the buriti fruit has aroused great interest in the food industry due to its high content of bioactive, including tocopherols, phenolic compounds, flavonoids, antioxidants, carotenoids (especially β-carotene) and monounsaturated fatty acids, such as oleic acid. This oil is one of the main sources of provitamins A and E, providing benefits such as antioxidant action, strengthening the immune system and healing.^48–52^

To date, no studies have been found that explore the clinical potential of OB byproducts in bone tissue. Therefore, with the aim of developing a material capable of performing the functions of bone tissue, we synthesized a porous, biocompatible and reproducible scaffold, with a continuous hierarchical porosity. For this, we use MAG as a soft segment, PEG 400 as a chain extender and hexamethylene diisocyanate (HDI) as a hard segment which, combined, can be applied directly to bone tissue.^16,53^ The hypothesis of our study is that the scaffold has biological potential to be a precursor in the synthesis of polymers for medical applications, since its biological properties are directly related to its chemical structures.

## Materials

Crude buriti oil was purchased in the southern region of the state of Piaui in the city of Sebastião Leal (7° 33′ 57″ S 44° 03′ 50″ W) located 400 km from Teresina. Glycerol, lithium hydroxide (LiOH), were purchased from Merck, India. HDI was obtained from Sigma-Aldrich and PEG 400 was purchased from SD Fine Chemicals. All chemicals were used as received, without any modifications.

## Synthesis

The synthesis of scaffolds is a two-step process. As oleic acid TAGs do not contain free hydroxyl groups, the first step consists of the formation of a polyol, MAG, from OB. The second step involves the reaction between MAG, hexamethylene diisocyanate and PEG 400 to produce the scaffolds described in this work.

### Step 1: Synthesis of MAG from Buriti Oil

MAG was synthesized using the hydrolysis method catalyzed by a strong base, as described in the literature, with some modifications.^40^ The OB and glycerol were simultaneously added to a beaker in a molar ratio of 1:3 (OB/glycerol) keeping the mixture under constant stirring for 10 min at 70.0 ± 5 °C to homogenize the system. Then 0.05% (mLiOH/mOB) of LiOH was added to the reaction and it was kept under constant stirring for 4 h. The reaction mixture was transferred to a separation funnel and presents two phases, the densest phase being MAG (Figure 1).

**Figure 1:**
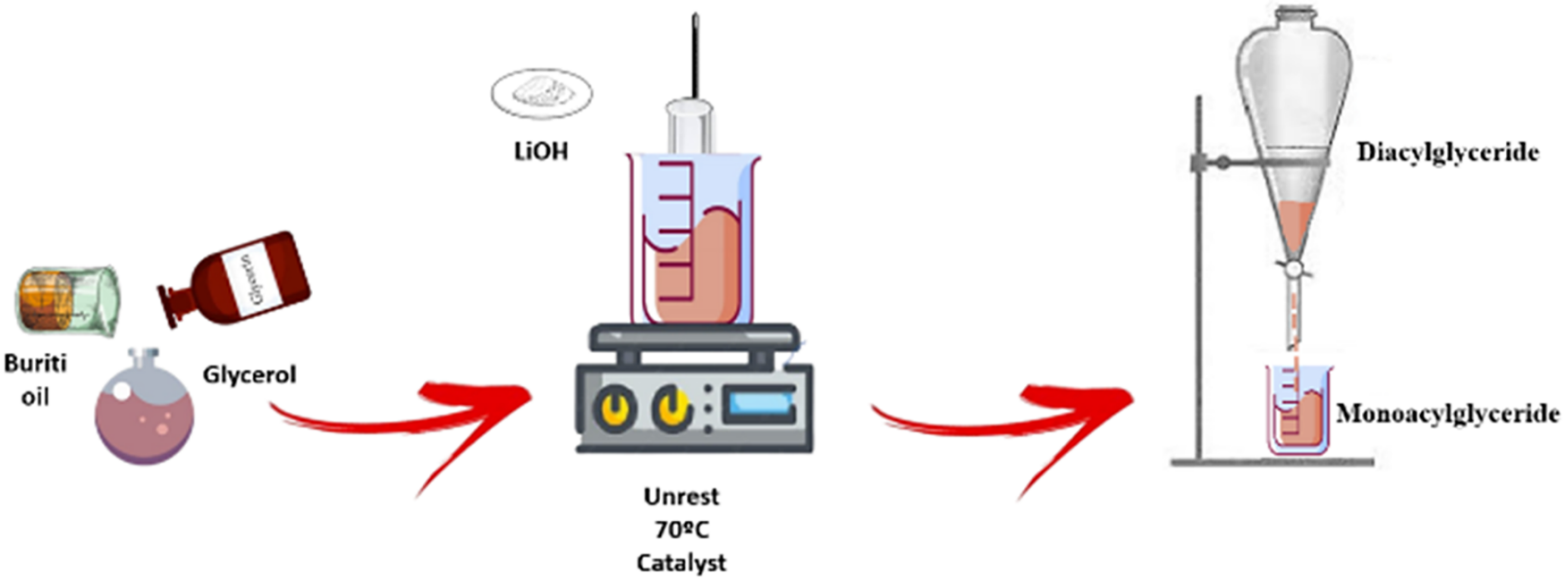
Scheme of monoacylglyceride synthesis.

### Step 2: Scaffold Synthesis

The scaffolds were synthesized by reacting MAG and PEG 400, catalyzed by HDI. Initially, after stabilizing the temperature, MAG (0.75 g), prepared in step 1, and PEG 400 (0.95 g) were added to a beaker and stirred for 10 min, until the system was homogenized. Subsequently, HDI (2.0 mL) was added to the mixture. The reaction mixture (MAG, PEG 400 and HDI) was stirred for 40 min at 65.0 ± 5 °C in an open system, observing an increase in viscosity and the formation of bubbles. The reaction product was cooled to room temperature (Figure 2), obtaining the scaffolds (Sc1). The amount of HDI for reactions with different amounts of PEG 400 was set at 2 mL. A similar procedure was applied to obtain a scaffold with a lower PEG content (0.85 g, labeled as Sc2) using 0.75 g of MAG.

**Figure 2:**
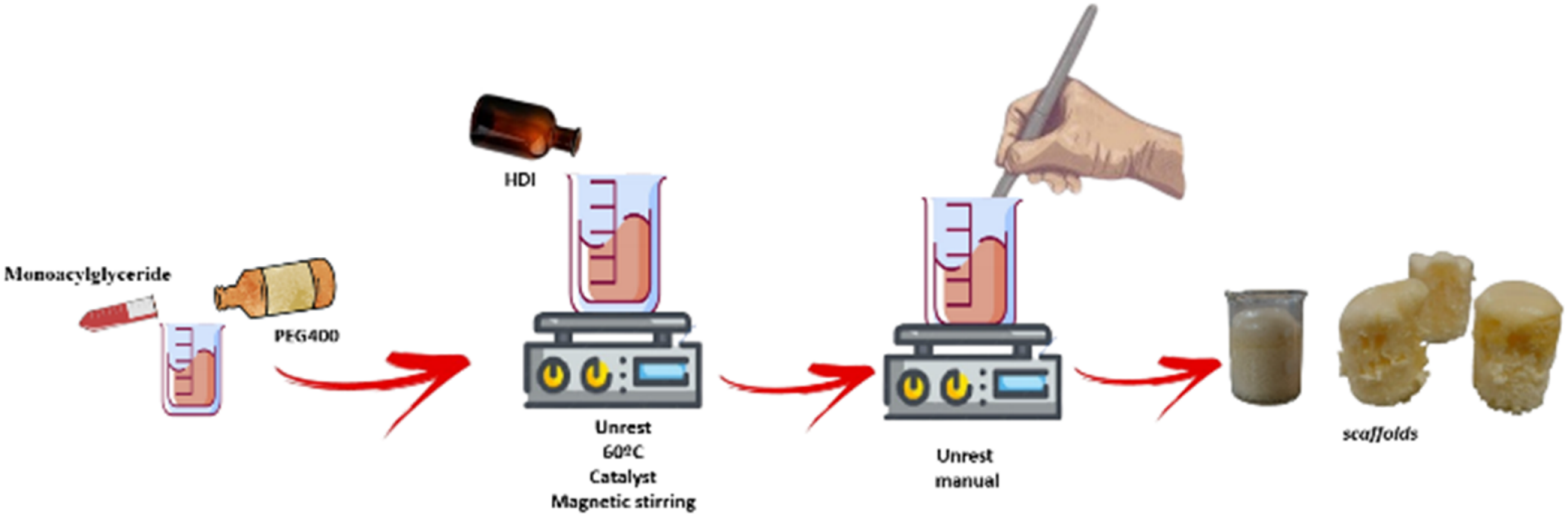
Scheme of scaffold synthesis.

### Characterization of Materials

Fourier-transform infrared (FTIR) spectra of monoacylglyceride, PEG-400, hexamethylene diisocyanate (HDI), and the synthesized scaffold (Sc1) were acquired using a Bruker Vertex 70 spectrometer equipped with an attenuated total reflectance (ATR) accessory. Spectra were recorded in the range of 400–4000 cm^–1^, with a spectral resolution of 4 cm^–1^, averaging 64 scans per sample. All measurements were obtained in absorbance mode.

Ultraviolet–visible (UV–vis) absorption spectra of the triacylglyceride (TAG) extracted from Mauritia flexuosa (buriti oil) and the scaffold (Sc1) were collected using a Shimadzu UV-2600 spectrophotometer. Samples were prepared by diluting 5 μL of the solution in 3 mL of dimethyl sulfoxide (DMSO) and analyzed in a 3.5 mL quartz cuvette. The same procedure was applied to both samples. Spectra were recorded in the wavelength range of 185–900 nm in absorbance mode.

Scanning electron microscopy (SEM) micrographs of the scaffolds Sc1 and Sc2 were acquired using a field emission gun scanning electron microscope (FEG-SEM), FEI Quanta FEG 250, operated at an accelerating voltage ranging from 1 to 30 kV. The equipment is coupled with an energy-dispersive X-ray spectroscopy (EDS) system (Bruker Quantax EDS) featuring an XFlash 5010 silicon drift detector (SDD). Pore size distribution and average pore diameter were estimated from SEM images using ImageJ software. For each sample, measurements were taken from at least 90 distinct points to ensure statistical relevance.

Thermogravimetric analysis (TGA) was performed using a Netzsch STA 449 F3 Jupiter simultaneous thermal analyzer. Approximately [insert sample mass if known] of each scaffold was placed in an alumina (Al_2_O_3_) crucible. The analysis was carried out from 50 to 600 °C at a constant heating rate of 25 °C/min. Nitrogen (N_2_) was employed as both purge gas (50 mL/min) and protective gas (20 mL/min) to maintain an inert atmosphere during the measurements.

The mechanical properties of the scaffolds were assessed by compression testing. Cylindrical specimens (56 mm in diameter × 23 mm in height) were prepared for both groups. Tests were performed using a mechanical testing machine (NanoMec50, HSensor) equipped with a 50 N load cell at a deformation rate of 3 mm·min^–1^. Data normality was evaluated using the Kolmogorov–Smirnov test, followed by Student’s *t* test. Statistical significance was set at *p* < 0.05. All analyses were performed with OriginPro 2018.

The swelling capacity was determined by measuring the initial dry weight of the scaffold and its wet weight after incubation. Initially, the samples were weighed and then immersed in 20 mL of distilled water maintained at 37 °C. Immersion periods of 1, 3, 5, and 7 days were applied. At each predetermined time point, the scaffolds were removed from the solution, gently blotted with filter paper to remove excess surface water, and subsequently weighed. The swelling ratio (SR) was calculated according to eq 1.^54^

1 $$\mathrm{Swelling}\left(\%\right)=\frac{W_{w}-W_{d}}{W_{d}}\times 100$$

*W*_w_ represents the weight of the sample in the swollen state, whereas *W*_d_ corresponds to the weight of the sample in the completely dehydrated state.

For the in vitro degradation study, PU samples were cut into dimensions of approximately 10 mm × 5 mm × 2 mm, immersed in 10 mL of phosphate-buffered saline (PBS, pH 7.2), and incubated at 37 °C for 28 days. At intervals of 7 days, the samples were removed from the solution, and residual surface water was carefully removed using absorbent paper prior to mass measurement. The samples were then dried at 60 ± 5 °C, and their dry weight was recorded. The percentage of remaining mass was calculated according to eq 2.^55^

2 $$\mathrm{Weight}\;\mathrm{loss}=\frac{W_{0}-W_{t}}{W_{0}}\times 100$$

where *W*_0_ is the initial dry weight and *W_t_* is the dry weight at time *t*.

#### Cytotoxicity Assays

To evaluate if scaffolds have any cytotoxic effects on the rat bone-marrow derived mesenchymal stem cells (BMMSC), the 3-(4,5-dimethylthiazol-2-yl)-2,5-diphenyltetrazolium bromide (MTT) assay was adapted from the protocol described by Capella et al.,^56^ which evaluates cellular viability based on mitochondrial function through the reduction of MTT to a colored insoluble formazan salt. The BMMSC were seeded at 1.31 × 103 cells per well in a 96-well plate, cultivated in 100 μL D-MEM 15% FBS for 24 h. Next, samples of the Sc1 and Sc2 scaffolds were placed over the culture and incubated for 24, 48, and 72 h. After that, the scaffolds were removed, and the MTT-assay was carried out. A control (BMMSC in usual culture medium) was included to validate the viability protocol. Afterward, at each time point, the wells were rinsed with PBS buffer, and a MTT solution (0.5 mg mL^–1^) was added and incubated for 4 h. The solution was removed, and 100 μL was used to dissolve the formazan crystals. The absorbance of colorants was recorded at 570 nm in a microplate reader (Biotek Elx 800, Winooski, VT). Cytotoxicity was expressed as OD (optical density) of formazan. Statistical analyses were performed by two-way ANOVA with GraphPadPrism 8 software for statistical computing. Posthoc comparisons were performed using Dunett’s posthoc test. The values are expressed as mean ± standard error (S.E.) and were considered significantly when *p* ≤ 0.05. For * (*p* < 0.05), ** (*p* < 0.01), *** (*p* < 0.001) and **** (*p* < 0.0001).

3 $$\mathrm{Formula}:\left[\%\right]=\frac{\mathrm{pellet mass}\left(g\right)}{\mathrm{solution volume}\left(\mathrm{mL}\right)}\times 100$$

To obtain an extraction solution for the biomaterials, weighing was performed followed by the addition of saline solution to obtain a concentration of 2 mg mL^–1^ and incubated at 37 °C for 1 h. Then, 800 μL of the extraction solution was mixed with 200 μL of the 2% red blood cell solution in Eppendorf tubes. For the negative control, 800 μL of saline solution was used, for the positive control, 800 μL of distilled water. The tubes were shaken and incubated at 37 °C for 1 h; the test was performed in triplicate. After the incubation time, the tubes were centrifuged at 3.000 rpm for 10 min, the supernatant liquid was collected with a micropipette and taken for analysis in the spectrophotometer at 545 nm (DU 800 UV/Visible Spectrophotometer, Beckman Coulter). After reading, the percentage of hemolysis was obtained using the formula [Disp-formula eq4]

4 $$\%H=\frac{\mathrm{AB}-\mathrm{AS}}{\mathrm{AA}-\mathrm{AS}}\times 100$$

Where AB is the absorbance of the experimental tube, AS is the absorbance of the negative control (saline) and AA is the absorbance of the positive control.

## Results and Discussion

To preserve the properties of OB, the chemical modification in TAG to obtain MAG was limited to breaking only two fatty acid chains, keeping the glycerol portion and one of the TAG chains intact. The polyol (MAG) used as a monomer for polyurethane synthesis was prepared from the glycerolysis reaction using LiOH as a catalyst. This process occurred at low temperature (70.0 ± 5 °C) to avoid the generation of undesirable byproducts, such as acrolein. In addition to the change in color and odor of the product. The synthesis (Figure 3) shows the mechanism of MAG formation. Initially, the deprotonation of a hydroxyl group of glycerol by LiOH occurs, forming the alkoxide^57,58^ Therefore, the reaction takes place without the need for a catalyst. The alkoxide acts as a nucleophile, reacting with the carbonyl of TAG, forming an unstable intermediate, which rapidly decomposes to release an alcohol (usually a diacylglyceride (DAG) or a free fatty acid). Then another nucleophilic attack occurs on the carbonyl of the DAG formed, producing MAG.

**Figure 3:**
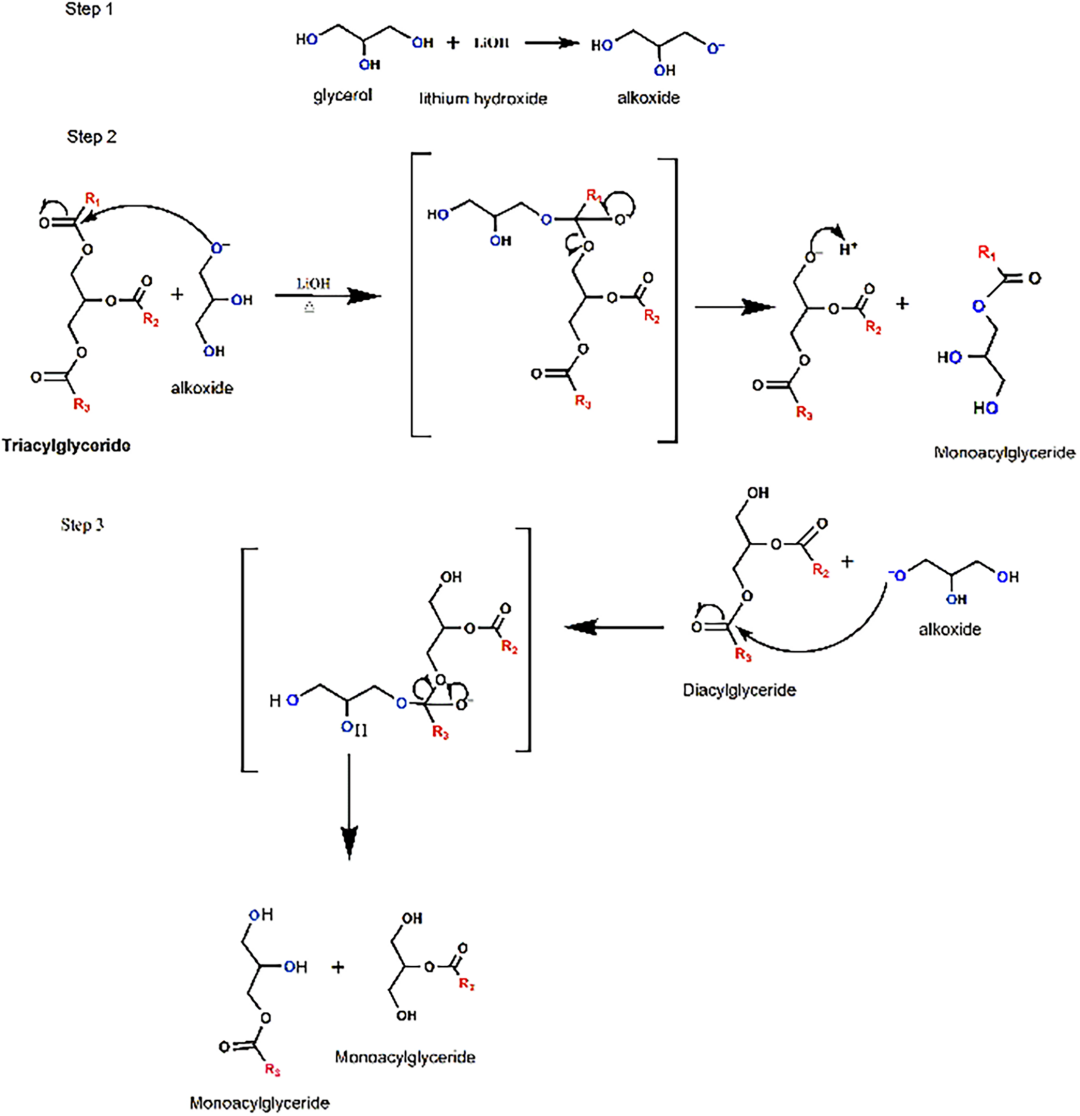
Mechanism of monoacylglyceride synthesis.

The synthesis (Figure 4) shows the mechanism of scaffold formation. Similarly, both the free hydroxyl group of the MAG of oleic acid and the nucleophilic oxygen of the hydroxyl group in PEG 400 attack the electrophilic carbon of the isocyanate group of HDI, forming a urethane bond, which results in a three-dimensional polymer network integrating both hydrophilic (PEG) and hydrophobic (MAG) segments. In this reaction, HDI was used as a catalyst and PEG 400 as a chain extender.

**Figure 4:**
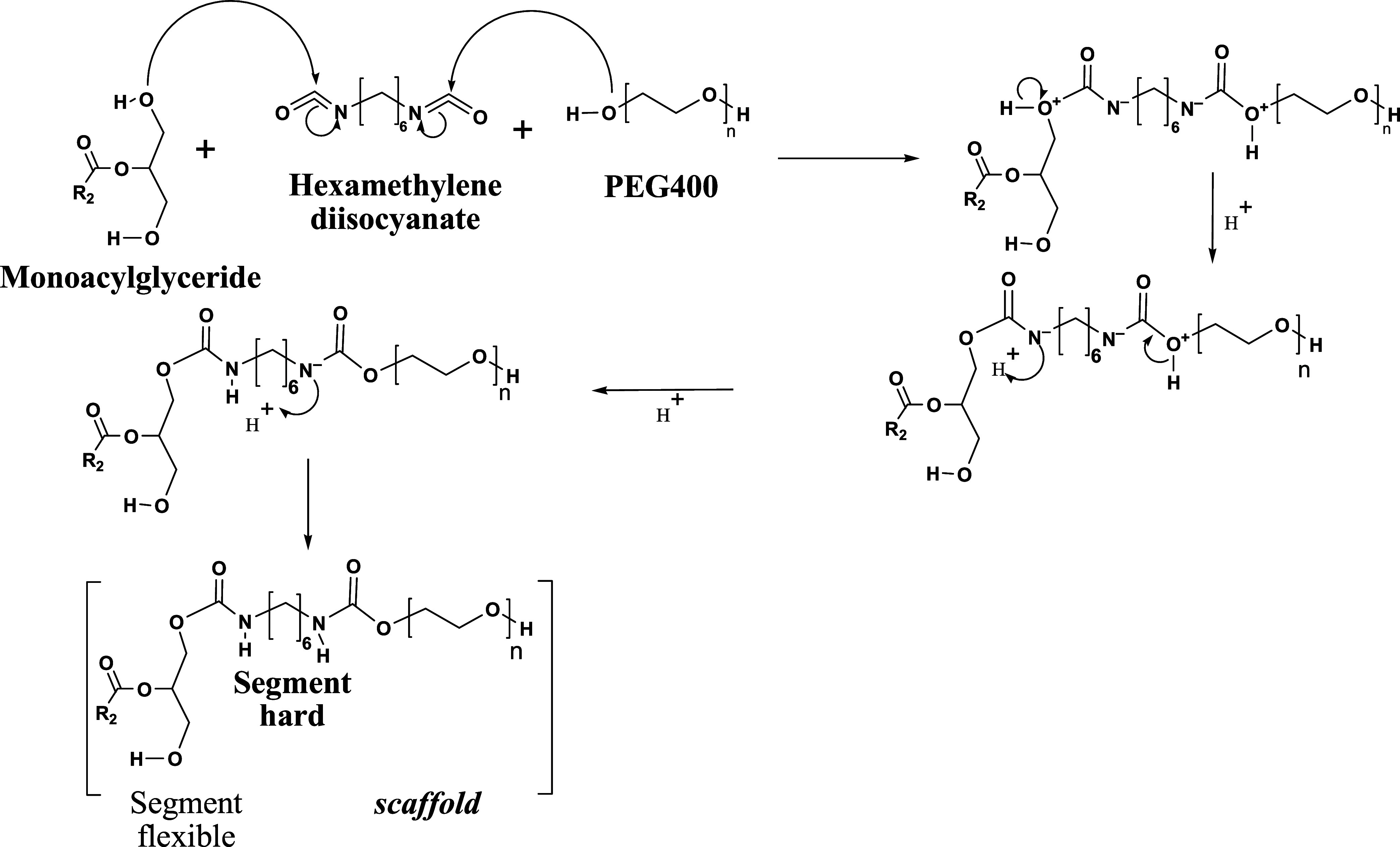
Mechanism of scaffold synthesis.

### Structural Analysis

Figure 5 displays the FTIR spectra of MAG, PEG 400, HDI, and the scaffold (SC1), highlighting the characteristic vibrational absorption bands of each component. In the MAG spectrum, obtained from the reaction between glycerol and buriti oil, a broad band at 3323 cm^–1^ is observed, attributed to the hydroxyl group from glycerol—absent in buriti oil—and a band at 1745 cm^–1^, corresponding to the ester carbonyl (C=O) stretching, present in buriti oil but not in glycerol. The PEG 400 spectrum exhibits two prominent bands: one at 3455 cm^–1^, assigned to the hydroxyl (−OH) stretching, and a more intense band at 1092 cm^–1^, associated with the asymmetric stretching of the −C–O–C– ether group. The HDI spectrum shows a strong absorption at 2261 cm^–1^, characteristic of the isocyanate (−N=C=O) stretching vibration.^58^

**Figure 5:**
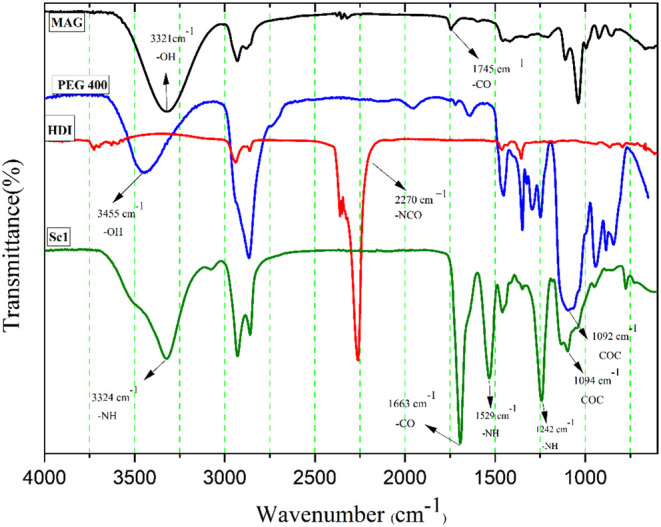
Infrared spectrum of MAG, PEG-400, HDI, and scaffold (Sc1).

The Sc1 scaffold spectrum reveals distinct bands confirming urethane bond formation, including a broad and intense band at approximately 3324 cm^–1^ (N–H stretching), as well as bands at 1663 cm^–1^ (C=O, amide I), 1528 cm^–1^ (N–H bending, amide II), and 1242 cm^–1^ (C–N stretching, amide III). Additionally, the band observed at 1094 cm^–1^ is attributed to the −C–O–C– stretching, confirming the incorporation of PEG into the polymer matrix.^59^

The absence of the band at 2261 cm^–1^, corresponding to the isocyanate (−N=C=O) stretching, indicates the complete consumption of isocyanate groups, confirming the successful formation of the polyurethane network.^34,60–63^

### UV–visible

Buriti oil is recognized for its diverse biological properties, attributed to its bioactive compounds, particularly carotenoids, with β-carotene being the predominant carotenoid. Additionally, buriti oil exhibits significant antifungal, anti-inflammatory, antibacterial, and antioxidant activities.^1,2^ To ensure the effective preservation of these bioactive compounds, it is essential to maintain their stability within Sc1 and Sc2, as confirmed by UV–vis spectroscopy.

The UV–vis absorption spectra of TAG and the scaffolds are presented in Figure 6. Absorption is observed in the range of 250 to 550 nm, indicative of carotenoid presence. It is noteworthy that although fatty acids and their oxidative derivatives present in the oil show absorption in the 250–350 nm range, these absorptions are associated with forbidden *n* → π* transitions (carbonyl groups) and π → π* transitions (nonconjugated double bonds), with peak absorption typically occurring around 200 nm.^64^

**Figure 6:**
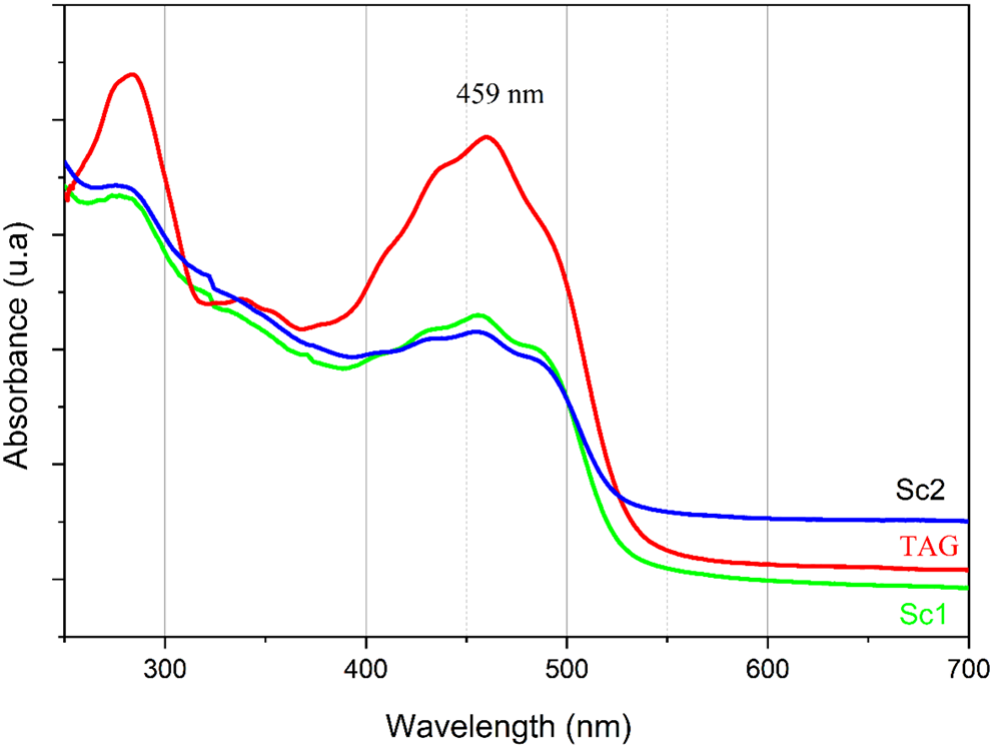
UV–vis absorption spectra (−) Buriti oil (TAG),(−) scaffold (Sc1) and (−) scaffold (Sc2).

In contrast, β-carotene possesses a conjugated system, where π → π* transitions occur at longer wavelengths due to the closer energy levels of the molecular orbitals. Consequently, the absorption observed at 459 nm in the oil is attributed to its β-carotene content.^65^ Although the intensity is lower, the spectra of Sc1 and Sc2 also show a band at 459 nm, confirming the presence of this bioactive compound. This reduced intensity is likely a result of changes in the concentration of β-carotene due to chemical processing, including reactions with glycerol, HDI, and PEG, which can alter the absorption intensity and spectral pattern in this region.^66^

These findings suggest that the scaffolds represent a promising alternative for the development of innovative products with potential health benefits, demonstrating their viability in applications requiring bioactive compound retention.^67^

### SEM Analysis

The surface and cross-sectional morphologies of the scaffolds are shown in Figure 7. All samples present high porosity and uniform pores. The average pore sizes observed in Sc1 and Sc2 are 258 ± 106 and 248 ± 110 μm, respectively, exhibiting an open pore structure with predominant connectivity and a uniform pore distribution. For significant bone formation it is essential to consider pore size, as newly formed blood vessels, which supply sufficient oxygen and nutrients for osteoblastic activity, require pores in the range of 200–350 μm for osteoblast proliferation.^68–72^

**Figure 7:**
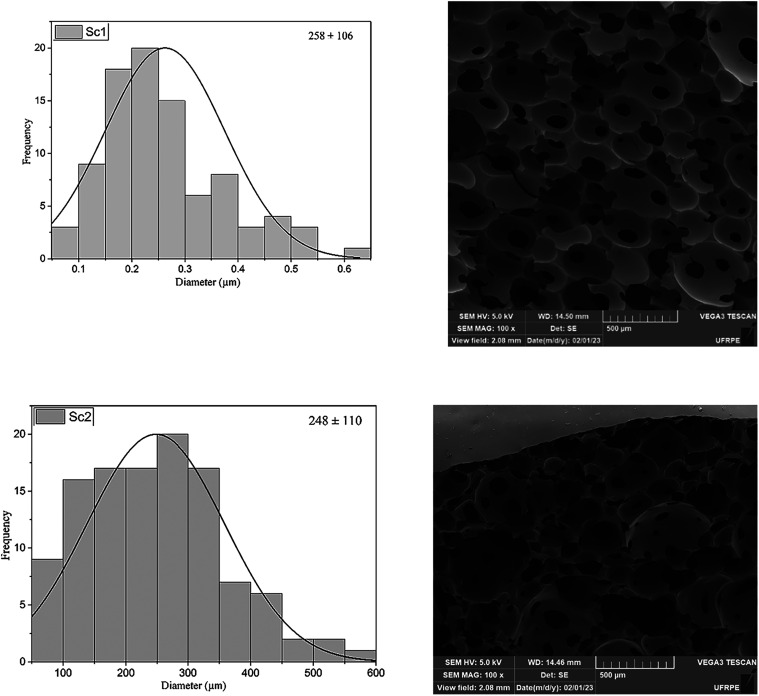
Pore size distribution in synthesized scaffolds and SEM micrographs of Sc1 and Sc2 scaffolds.

The scaffolds in question have concave surfaces, these surfaces create microenvironments that can increase cell adhesion, biochemical signaling, critical factors for cell proliferation and differentiation. Additionally, concave surfaces can improve the retention of growth factors and nutrients, optimizing support for cell growth. Therefore, the use of scaffolds with concave surfaces represents a promising strategy for ET, contributing to significant advances in regenerative therapies and tissue repair.^73,74^

### Thermal Characterizations

The thermal analysis of scaffolds Sc1 and Sc2 (Figure 8) was performed using Thermogravimetric Analysis (TGA) and Derivative Thermogravimetry (DTG) to investigate the thermal degradation stages and evaluate the thermal stability of these materials. In general, the thermal stability of these polyurethanes (PU) is closely related to the density of urethane groups per unit volume, which form the rigid segments (NCO) within the scaffolds.^75^ Since both scaffolds differ only in the amount of PEG 400 while using equivalent amounts of HDI and monoacylglyceride in the synthesis, similar degradation stages were observed.

**Figure 8:**
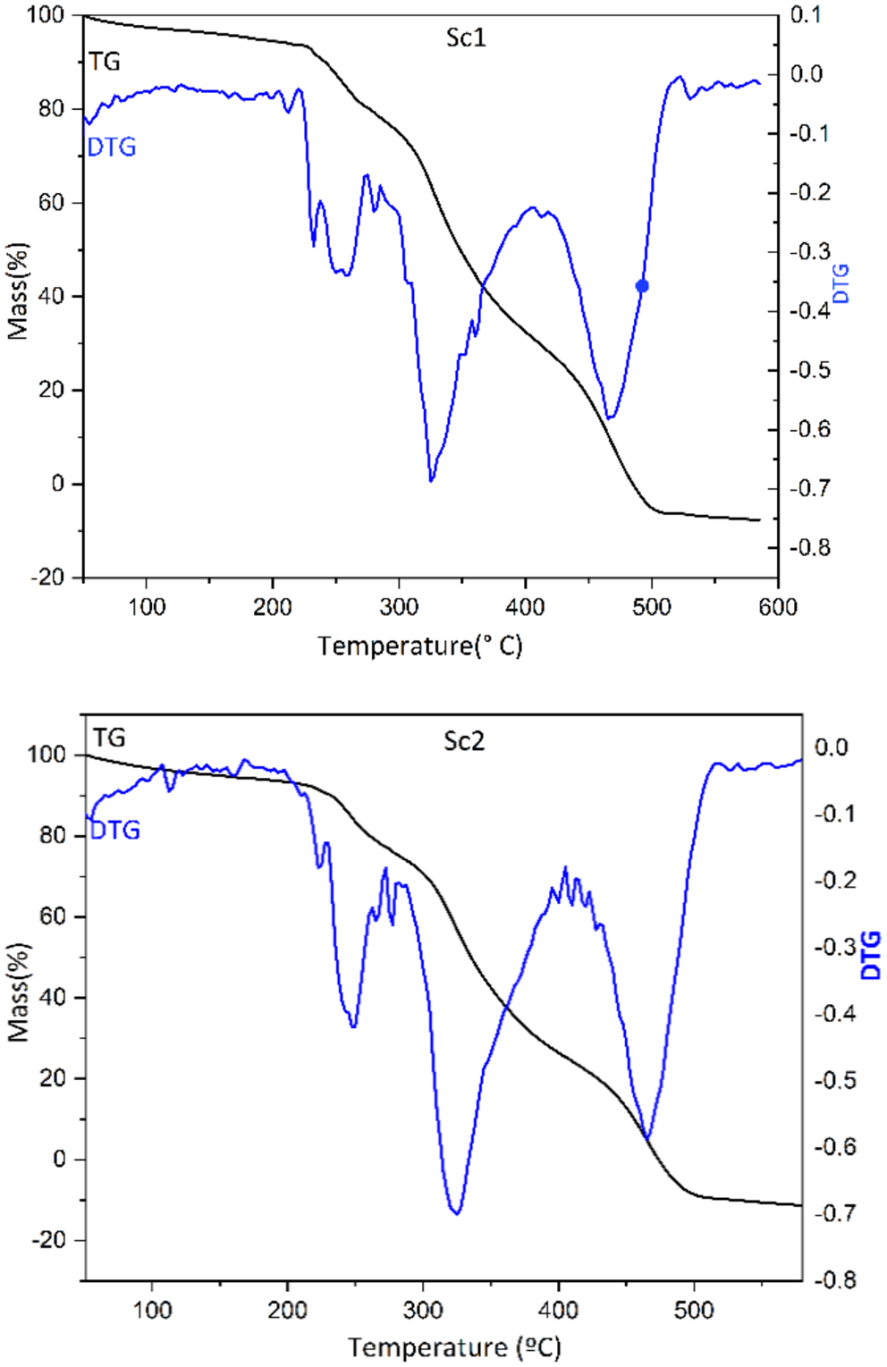
TGA and DTGA of materials of scaffolds Sc1 and Sc2.

An initial mass loss, ranging between 1% and approximately 7%, was observed in the TGA and DTG curves up to 225 °C, with Sc1 exhibiting a higher loss (∼7%) compared to Sc2 (∼5%). This is likely attributed to the physical desorption of volatile organic compounds, such as gases and moisture, which is enhanced by the higher PEG 400 content in SC1, due to its hygroscopic nature that favors water absorption.^68^

Both scaffolds exhibited three distinct degradation stages, occurring between approximately 225 and 500 °C. The first degradation stage, from 225 to 305 °C, resulted in a mass loss of approximately 20%, associated with the decomposition of urethane linkages, leading to the dissociation of the scaffolds into isocyanates, alcohols, primary amines, terminal olefinic groups from polyester chains, and CO_2_.^76–78^

The second stage, occurring between 305 and 430 °C, is related to the degradation of the flexible segments, including the breakdown of ester bonds, dehydrogenation, and polycondensation of alkyl groups present in the MAG and PEG400 chains. This stage resulted in a mass loss of approximately 52% for SC1 and 50% for SC2. The DTG curves for both scaffolds exhibited a peak at approximately 325 °C, indicating the maximum decomposition rate. This behavior is mainly attributed to the higher PEG400 fraction, whose polyether chains display lower thermal stability compared to the rigid urethane-derived segments.^58,79^

The third degradation stage occurred at temperatures above 430 °C and is associated with the cleavage of C–C bonds or the decomposition of residual flexible fragments within the polymer structure. The degradation products in this stage depend on the nature of the polyurethane and correspond to the breakdown of advanced fragments formed during the second stage, including the presence of terminal olefinic groups and decomposition of carbonaceous residues.^80–82^

Overall, both Sc1 and Sc2 exhibited thermal stability compatible with biomedical applications, including use in bone tissue engineering, since they can withstand temperatures significantly higher than those encountered under physiological conditions.

### Mechanical Testing and Analysis of Scaffolds

The mechanical test was conducted to determine the compressive strength of the scaffolds. The SC1 group exhibited a stress of 1088.28 kPa, while the SC2 group recorded 2493.79 kPa, as illustrated in Figure 9. This figure presents a comparative boxplot between SC1 and SC2, demonstrating that SC2 has significantly higher stress than SC1, along with greater variability in the obtained values. Statistical analysis indicated normalized means of 0.35456 for SC1 and 0.94804 for SC2, with t-values of 178.62 and 184.44, respectively, and *p* < 0.0001. These results confirm that SC2 exhibits a highly significant difference compared to the null hypothesis for *p* < 0.05.

**Figure 9:**
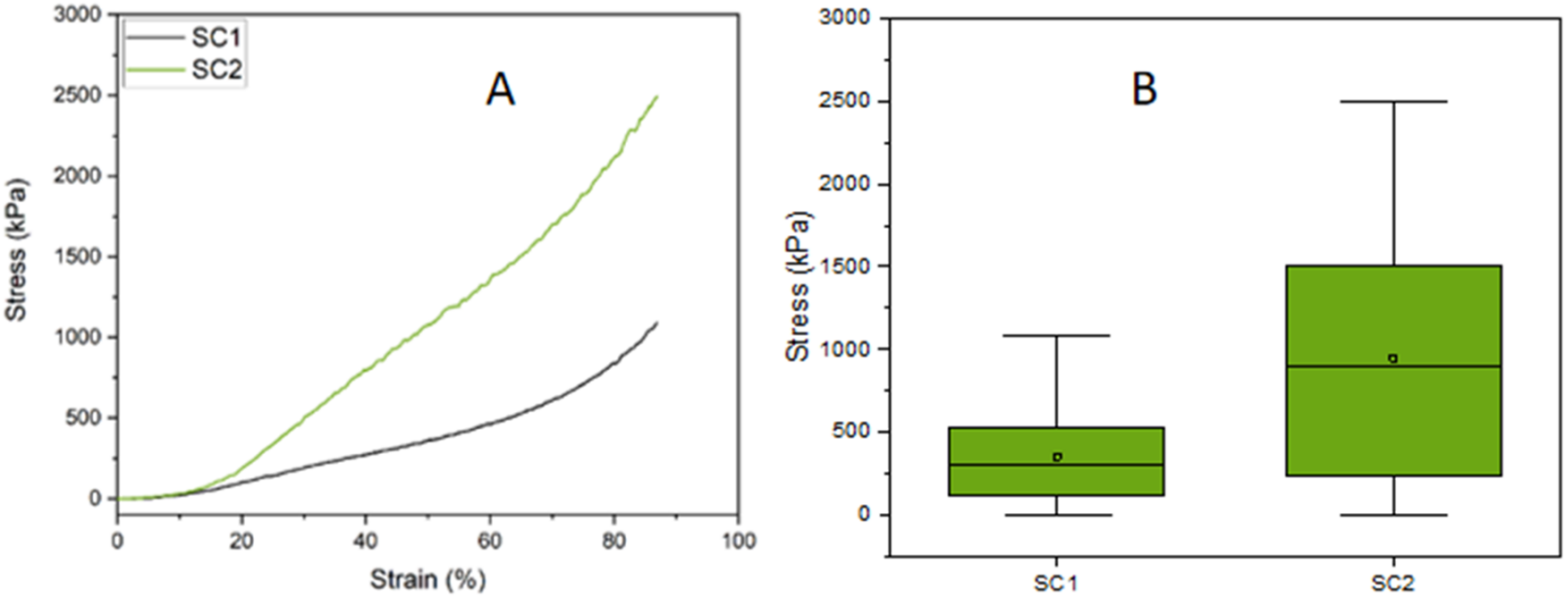
(a) Stress–strain curve for SC1 and SC2; (b) Comparative boxplot of stress in scaffolds from groups SC1 and SC2, with statistical analysis performed using the *t* test, showing a significant difference (*p* < 0.05) between SC2 and SC1.

The observed difference may be related to the relevant contribution of PEG400 concentration, which also influences scaffold pore formation and distribution. This finding aligns with the literature, which highlights that porosity and its structural characteristics are determining factors for the mechanical strength of spongy scaffolds.^83,84^ SEM micrographs (Figure 7) revealed predominantly open and uniformly distributed pores. In both groups, the average pore size was consistent with the mechanical results, considering that dimensions above 300 μm may compromise mechanical properties and hinder both nutrient transport and cell fixation.^83^

The addition of PEG400 may also promote interactions between material components through hydrogen bonding, enhancing structural mobility.^84^ This effect is noticeable in SC2, which exhibited higher fracture stress, suggesting a greater ability to withstand loads before significant deformation compared to SC1. These findings reinforce the importance of PEG400 in optimizing the mechanical properties of scaffolds. Thus, the combination of OB-derived MAG with PEG400 appears to be a promising strategy for developing scaffolds with potentially suitable performance for Bone Tissue Engineering (BTE) applications.^85^

### Swelling and Degradability

The swelling behavior of scaffolds reflects their fluid absorption capacity, directly influencing mechanical stability, nutrient diffusion, and cell adhesion during culture. Maintaining dimensional stability in an aqueous environment is essential for scaffolds intended for tissue regeneration, and hydrophilicity is crucial to ensure proper cell interaction without compromising structural integrity.^86^ Furthermore, the degradation rate should align with the tissue healing process to provide adequate support for new tissue formation. Rapid degradation may compromise structural support, whereas slow degradation can hinder natural regeneration.^87^

Based on these considerations, the swelling behavior of the scaffolds, driven by water uptake, was evaluated over 7 days, as shown in Figure 10a. Both scaffolds exhibited high water absorption, with swelling progressively increasing until equilibrium was reached on day 3 for SC1 and SC2. After 7 days, the average swelling values were 383.7 ± 10.1% for SC1 and 369.8 ± 10.9% for SC2, indicating that SC1 exhibited significantly higher swelling. This behavior is primarily attributed to pore size and porosity, as larger pores and higher porosity favor increased water absorption and influence degradation rate. Therefore, SC1 and SC2 demonstrated appropriate swelling properties, ensuring efficient fluid uptake and supporting optimized tissue regeneration (Figures 11 and 12).

**Figure 10:**
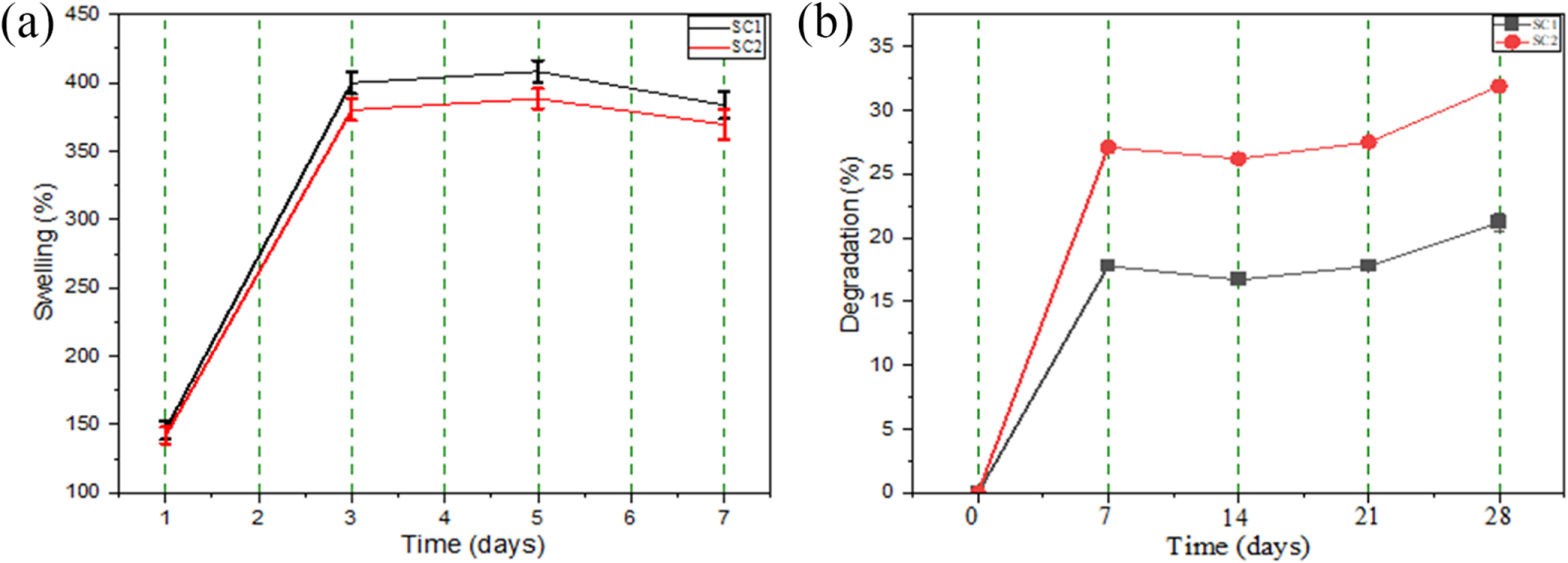
(a) swelling capacity of scaffolds SC1 and SC2 in distilled water over 7 days, (b) weight loss of scaffolds during 28 days of immersion in PBS.

**Figure 11:**
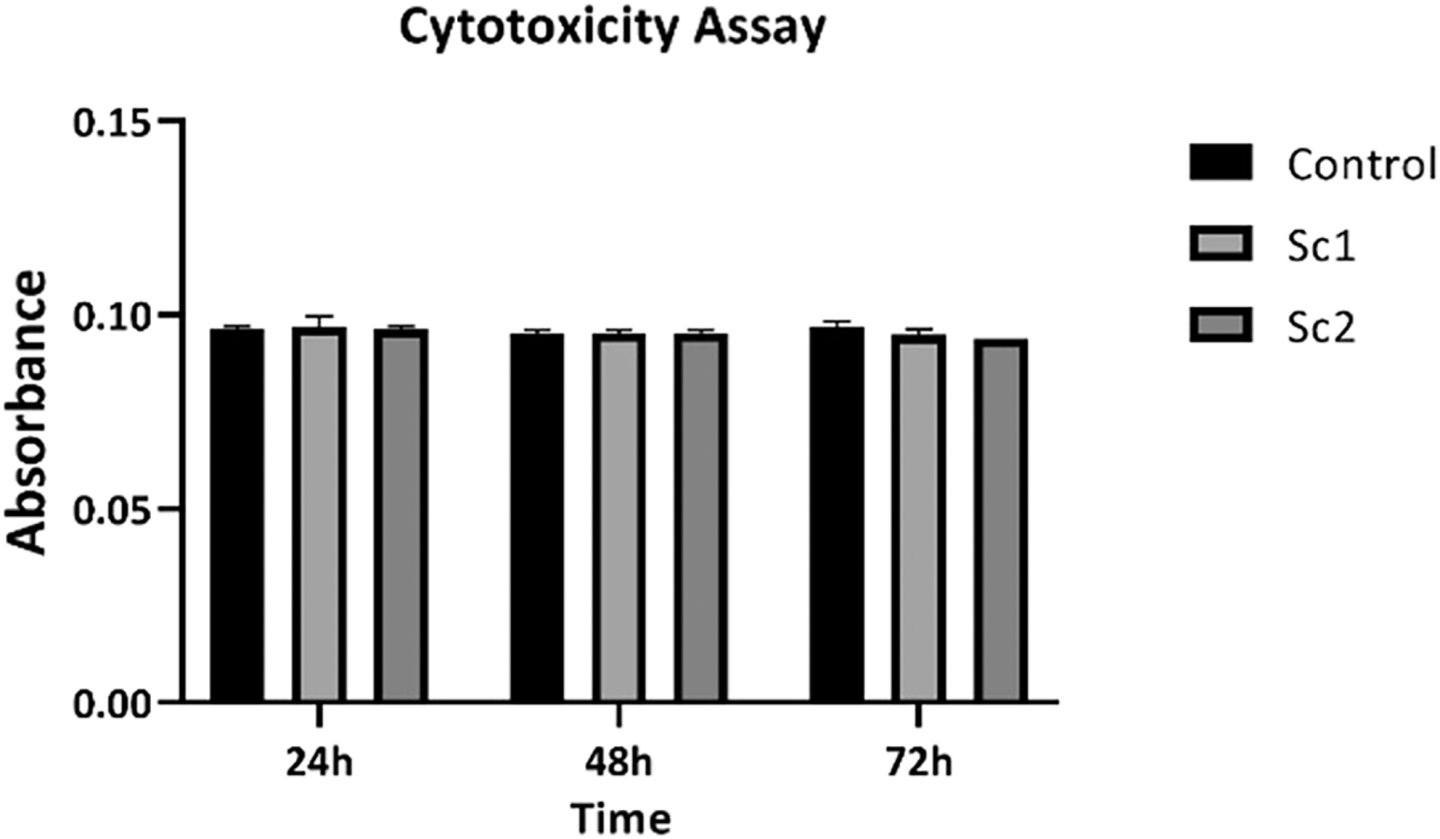
Cell viability graph of scaffolds (Sc1 and Sc2) by MTT assay.

**Figure 12:**
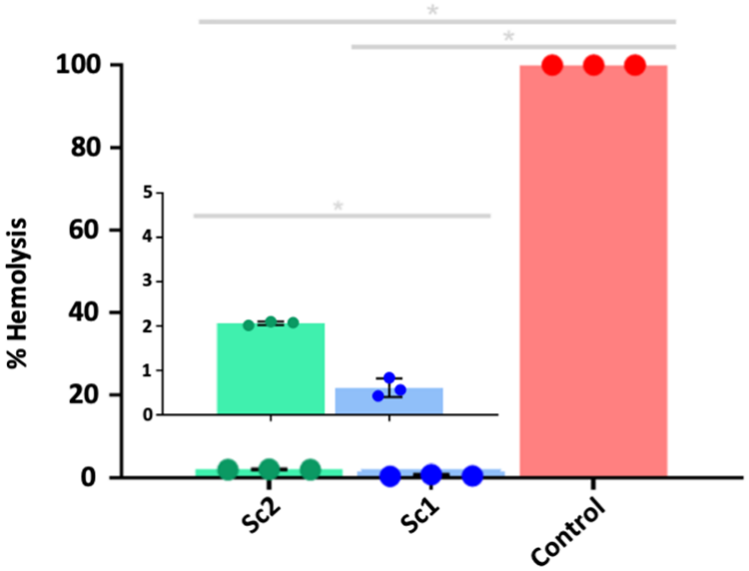
Hemocompatibility graph of Sc1, Sc2 scaffolds and control.

Scaffold degradation in PBS was monitored over 28 days, with results shown in Figure 5b. All formulations displayed consistent mass loss, with values of 31.21 ± 0.67% for SC1 and 31.83 ± 0.53% for SC2. This reduction is associated with polymer degradation, which involves fluid absorption, ester bond hydrolysis, and matrix erosion.^55^ Differences between SC1 and SC2 may be attributed to variations in porosity and PEG 400 content, which likely facilitated more efficient aqueous medium penetration and accelerated hydrolytic degradation.^88^

The results indicate that both SC1 and SC2 possess physicochemical properties suitable for tissue regeneration, combining high fluid absorption with a degradation rate compatible with healing time. Performance differences between the scaffolds are related to microstructural features, highlighting the importance of controlling pore size and porosity in scaffold development. SC1 exhibited slightly superior water absorption, suggesting greater potential to support tissue regeneration.

### MTT Viability

The MTT assay was used to evaluate cell viability after exposure of rat bone marrow-derived mesenchymal stem cells (BMMSC) for 24, 48, and 72 h. This time-dependent assay (Figure 9) revealed that the scaffolds do not have a cytotoxic impact. It was observed that the groups presented viability like the control at all times evaluated, and there may be an increase in cell proliferation due to the increase around cell adherence. It is believed that it may be directly related to the PEG 400 ratio due to its high hydrolificity.^89,90^ Based on this information, cell viability can be related to functional activity, which constitutes an important defense mechanism for human health.^47^

### Hemolytic Activity

The biocompatibility of the scaffolds was assessed using the hemocompatibility test, which has as its principle the assessment of damage to the plasma membrane of red blood cells and, consequently, the release of hemoglobin. (hemolysis). According to the Standard Practice for Evaluating Hemolytic Properties of Materials (ASTM-F756–08, 2000), samples with a percentage of hemolysis between 0–2% are classified as nonhemolytic, between 2–5%, slightly hemolytic and >5% are considered hemolytic. The hemocompatibility of the scaffolds was analyzed, in which the Sc2 material presented ≈2.0% hemolysis, while Sc1 presented <1.0 (≈2.0) % hemolysis, as demonstrated in Figure 10. It is observed that Sc1 has lower hemolytic activity than Sc2, this is attributed to the amount of PEG 400 that induces hydrophilicity of the material, contributing to biocompatibility.^47^ Considering that the stability of the blood clot is of fundamental importance for the tissue repair process of bone injuries, allowing the formation of granulation tissue that will lead to the formation of functional bone tissue, the high hemocompatibility demonstrates that buriti derivative scaffolds are safe and promising for use in bone ET.

## Conclusions

In this study, we developed scaffolds through the reaction between MAG, obtained from the glycerolysis of OB, and PEG 400, using HDI as a polymerizing agent, targeting its application in bone tissue engineering. The scanning electron microscopy (SEM) images revealed ideal porosity, essential for promoting cellular infiltration and vascularization, which are critical factors for bone regeneration. Furthermore, the scaffolds exhibited good thermal stability, crucial for maintaining structural integrity under physiological conditions. Biological tests, including hemolysis and MTT assays, showed that the scaffolds are biocompatible with low cytotoxicity, making them safe for contact with blood cells and tissues. These results suggest that the scaffolds possess adequate physical and chemical properties and are both effective and safe for biomedical applications, particularly in bone regeneration.
